# Staged, Open, No-Ischemia Nephron-Sparing Surgery for Bilateral-Multiple Kidney Tumors in a Patient with Birt-Hogg-Dubé Syndrome

**DOI:** 10.1155/2012/639629

**Published:** 2012-04-24

**Authors:** Ahmet Tefekli, Ayşe Deniz Akkaya, Kamil Peker, Terman Gümüş, Metin Vural, Fatin Cezayirli, Ahmet Musaoglu, Tarık Esen

**Affiliations:** ^1^Department of Urology, VKF American Hospital, 34365 Istanbul, Turkey; ^2^Department of Dermatology, VKF American Hospital, 34365 Istanbul, Turkey; ^3^Istanbul Pathology Group, 34365 Istanbul, Turkey; ^4^Department of Radiology, VKF American Hospital, 34365 Istanbul, Turkey; ^5^Koc University School of Medicine, 34365 Istanbul, Turkey

## Abstract

Hereditary kidney cancer patients with bilateral multiple kidney tumors represent challenges in the era of rapidly growing minimal invasive treatment techniques. Birt-Hogg-Dubé Syndrome (BHDS) is an autosomal dominant genodermatosis characterized by a triad of benign skin tumors (fibrofolliculomas, trichodiscomas, acrochordons) together with an increased risk of developing malignant renal tumors and pulmonary disease such as pneumothoraces and multiple lung cysts. The morbidity and mortality of the affected patients is determined by the presence of the kidney tumors, which tend to be multifocal and bilateral, as observed in other hereditary kidney cancer syndromes like von Hippel-Lindau disease, familial leiomyomatosis, and hereditary papillary renal cell carcinoma. Herein, a patient with BHDS, presenting with synchronous bilateral multiple kidney tumors, is reported. The report describes the management of kidney tumors with two-stage open nephron-sparing surgery in which the nonvascular clamping technique was utilized.

## 1. Introduction

Birt-Hogg-Dubé Syndrome (BHDS) is an autosomal dominantly inherited genodermatosis caused as a result of the mutations in the FLCN gene encoding the protein “folliculin,” a new protein which hypothetically functions as a tumor suppressor gene [1–4]. The affected patients develop cutaneous follicular tumors (multiple fibrofolliculomas, trichodiscomas, and acrochordons) over the face, neck, and upper trunk and are susceptible to develop renal neoplasms and pulmonary disease (spontaneous pneumothoraces, multiple lung cysts).

Kidney tumors in these patients tend to be multifocal and bilateral, as observed in other hereditary cancer syndromes such as von Hippel-Lindau disease, familial leiomyomatosis, and hereditary papillary renal cell carcinoma [5, 6]. Kidney tumors in BHDS are commonly and characteristically reported as “hybrid oncocytic renal cell carcinoma,” which contain areas of oncocytomas as well as areas of renal cell carcinoma, chromophobe, or others [4, 7]. Kidney tumors are the major determinants of morbidity and mortality in patients with BHDS; hence the overall renal functions have to be preserved as much as possible while completely removing the tumors within oncological principles [5]. Therefore, this syndrome may represent as an outstanding indication for open-staged nephron-sparing surgery (NSS), in the era of everyday growing laparoscopic/robot-assisted surgery [6–8].

**Figure 1:**
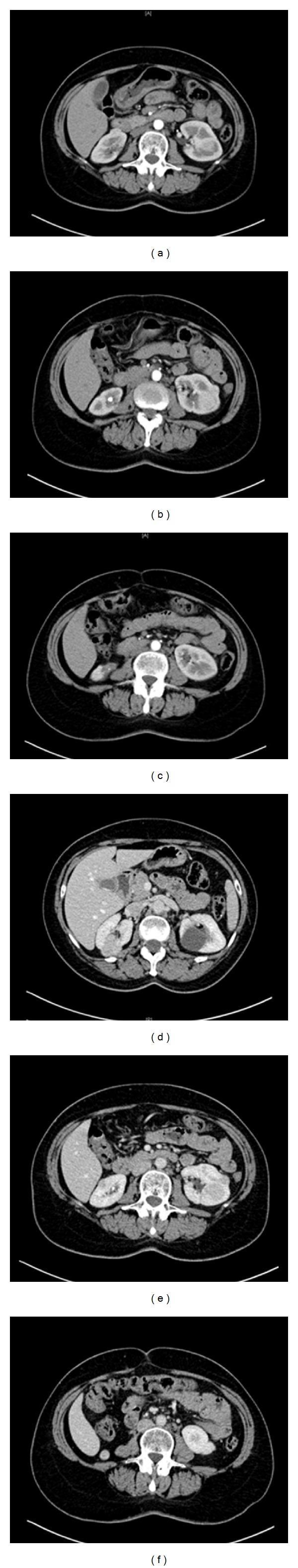
Preoperative contrasted CT scans of the patient, showing multiple bilateral kidney tumors, with diameters ranging between 1 and 5 cm. Radiologically, most of the tumors have malignant characteristics due to their hypervascularity and radiopaque enhancement.

**Figure 2:**
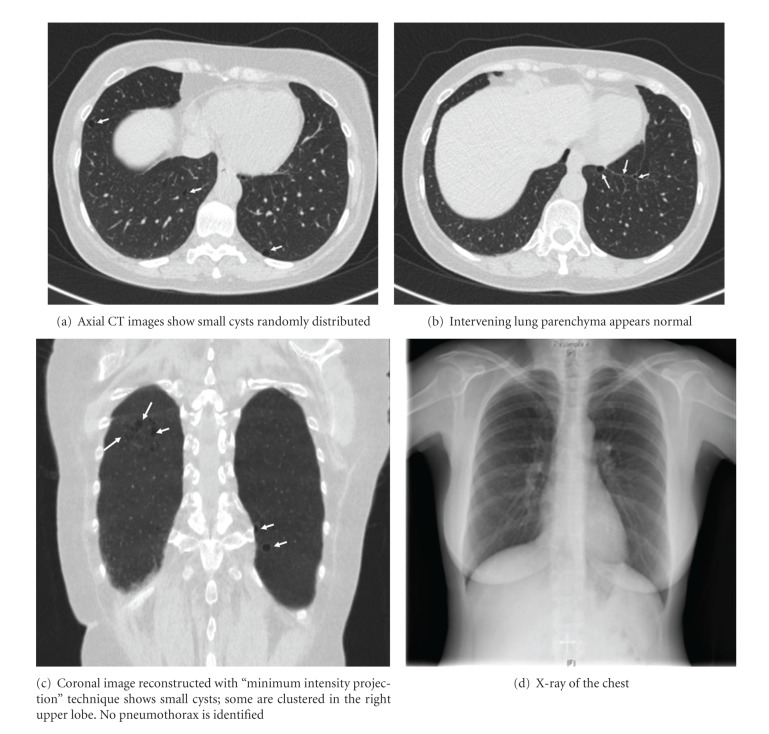
Preoperative CT scan of the thorax (a–c) and chest X-ray (d).

**Figure 3:**
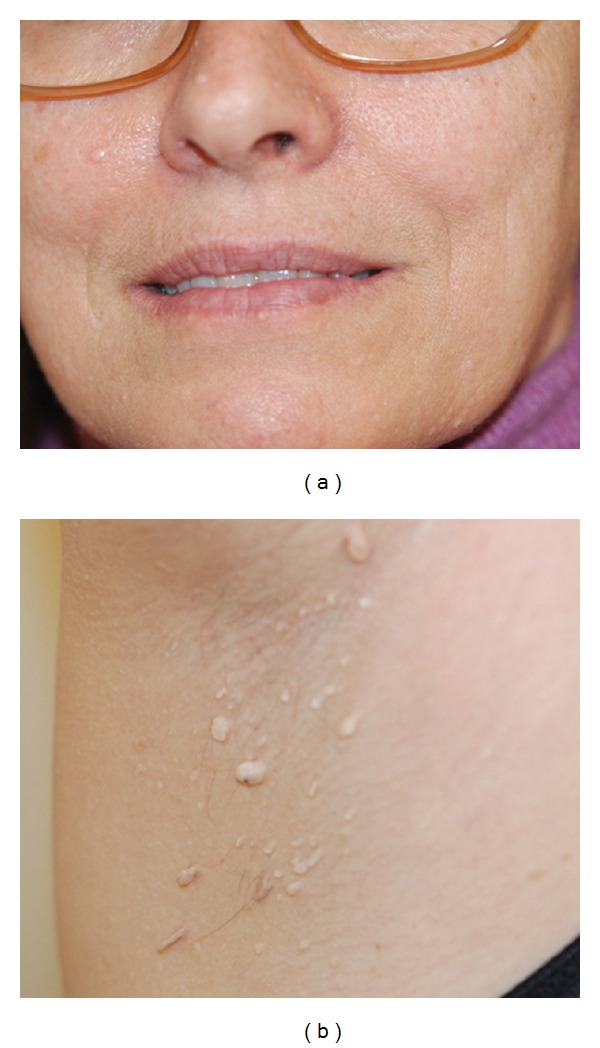
(a) Multiple, asymptomatic, white, dome-shaped, firm papules over the face. (b) Multiple skin colored, soft, pedunculated papules on the axillae (acrochordons).

We herein report a patient with BHDS, presenting with typical urological, dermatological, and radiological findings (Figures 1, 2, and 3). Kidney tumors of the patient were managed with two-staged open NSS, and we describe our no-ischemia surgical technique with the kidney under perfusion.

## 2. Case Report

A 56-year-old woman presented with bilateral multiple kidney tumors, diagnosed radiologically during her evaluation for hematuria.

Computed tomography revealed 4 tumors, diameters ranging between 1 and 3 cm in the left kidney, and 4 tumors, diameters ranging between 1 and 5 cm in the right kidney (Figure 1). Radiologically, most of the tumors had malignant characteristics due to their hypervascularity and radiopaque enhancement.

Her chest X-Ray and CT scan of thorax revealed multiple, randomly distributed air-filled small cysts (Figure 2). Intervening lung parenchyma appeared normal. Coronal images, reconstructed with “minimum intensity projection” technique, showed small cysts; most of them clustered in the right upper lobe. No pneumothorax was identified.

On dermatological examination, she had multiple, asymptomatic, white, 1–3 mm sized, dome-shaped, firm papules over the face, neck, scalp, upper trunk, and back, and multiple skin colored, soft, pedunculated papules on the axillae that were clinically consistent with BHDS (Figure 3). Her dermatological history revealed that her facial papules were present since her early twenties and continued to increase in number.

The clinical diagnosis of the patient was consistent with BHDS.

The patient was scheduled first for a staged approach starting from the right side. Preoperative serum analyses were within normal ranges (serum creatinine: 0.7 mg/dL, BUN: 13 mg/dL, Hb: 13.3 gr/dL), and the patient was healthy otherwise.

Nephron-sparing surgeries were performed using standard intercostals (between 11th-12th ribs) flank incision on each side. The technique consisted of adequate exposure of the kidney. Gerota's fascia was opened; perinephric fatty tissue was dissected off. Ureter and the vascular pedicle were exposed and marked with vessel loops. After documenting all tumors on the kidney, tumors were “enucleoresected” with the kidney under perfusion, first cauterizing the capsule surrounding the tumor and then by removing the tumor with the help of scissors and brain dissector. First the smaller tumors were enucleated, in order to diminish the risk of bleeding. Frozen sections from tumor beds were done for all tumors. Bleeding from the tumor bed was controlled with 3/0 polyglactin (Vicryl) interrupted sutures and parenchyma was adapted with 2/0 polyglactin (Vicryl) sutures over a surgical bolster. Renal vessels were neither clamped nor compressed during these manipulations.

A total of 4 tumors were enucleated from the right kidney. Operation time was 105 minutes. Time spent during tumor enucleations and renographies was 45 minutes. Estimated blood loss was 100 mL. Postoperative course was uneventful, with a postoperative serum creatinine of 0.7 mg/dL and serum Hb of 11.9 gr/dL. The patient was discharged 4 days after left NSS.

The left kidney was treated 6 weeks after the first stage. Using the same, open, no-ischemia NSS technique, a total of 4 tumors were enucleated from the left kidney. Operation time of the left side was 120 minutes. Time spent during tumor enucleations and renographies was 55 minutes. Estimated blood loss was 150 mL. Postoperative course was also uneventful on the right side and the patient was discharged 4 days after surgery.

**Figure 4:**
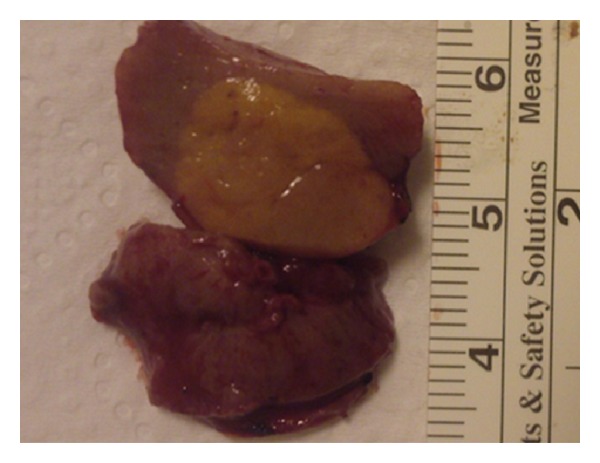
Macroscopic view of one of the tumors removed. The cut section appears light brown and glistering. No necrosis or hemorrhage is noted.

On macroscopic examination (Figure 4), the excised tumors of the right kidney measured 3.7 cm, 1.8 cm, 1.7 cm, and 1.2 cm in maximal diameter, respectively. The cut sections of each tumor appeared light brown and glistening. No necrosis or hemorrhage was noted. Excised tumors of the left kidney measured 2.7 cm, 2.1 cm, 1.8 cm, and 0.5 cm in maximal diameter, respectively. Cut sections of each tumor were the same with those of right kidney. All of the tumors appeared sharply circumscribed.

**Figure 5:**
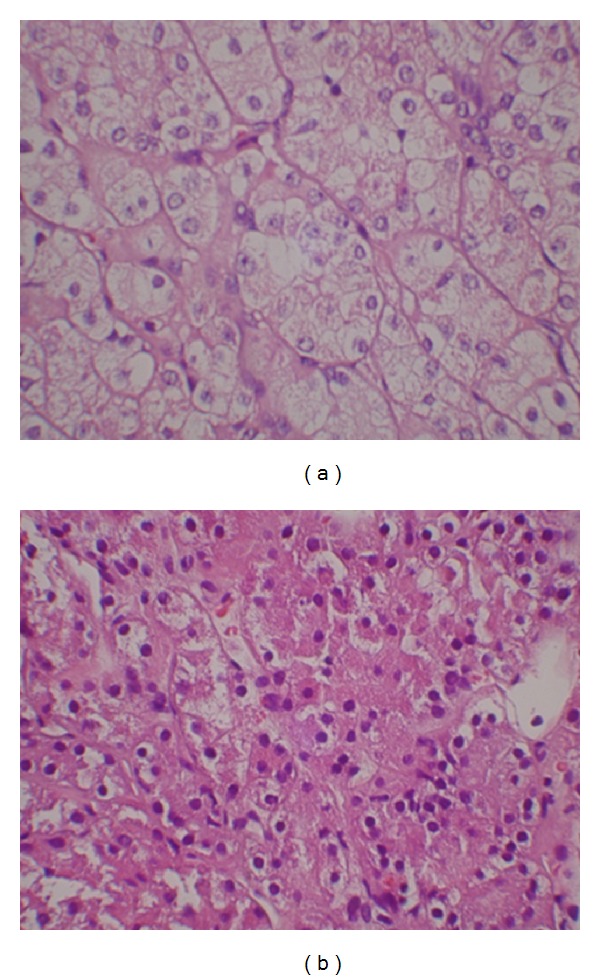
Microscopic views of H&E sections, demonstrating histological features of both oncocytoma and conventional variant of renal cell carcinoma.

Histomorphologically, each tumor revealed similar features (Figure 5). The H&E sections demonstrated histological features of both oncocytoma and conventional variant of renal cell carcinoma (clear cell RCC). Within these hybrid oncocytic tumors were areas classic for oncocytoma (large cells, ill-defined cell borders, finely granular eosinophilic cytoplasm throughout, and large nuclei with homogeneous basophilic chromatin) and other areas consistent with clear cell RCC (well-defined cell borders, clear cytoplasm, round nuclei with centrally located inconspicuous nucleoli). Fuhrman nuclear grade was II in clear cell RCC component. Mitotic features were sparse; no atypical forms were identified. Surgical margins were all negative.

**Figure 6:**
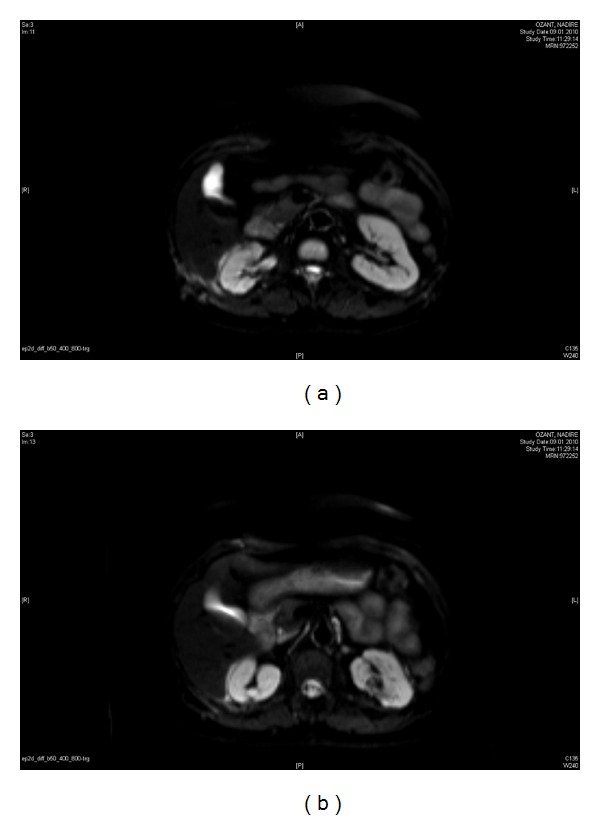
MRI scans performed 2 years after surgery, documenting no tumoral lesion.

The final serum creatinin level, seen 2 years after the last operation, was 0.9 mg/dL, and her abdominal MR scans (Figure 6) did not show any recurrences or new tumor formation.

## 3. Discussion

Partial nephrectomy, or “NSS,” has become the standard procedure for management of small renal tumors [9, 10]. However, there is a debate going on regarding the technique of applying renal ischemia during NSS to avoid renal damage in the treated kidney [10]. Current literature supports safe ischemia times, within 20 minutes of warm ischemia and up to 2 hours of cold ischemia, to minimize renal ischemic damage. Thus, if ischemia is required, the tumor should be removed within 20 minutes of warm ischemia, regardless of surgical approach, either open or laparoscopic [10].

However, most of the data regarding our current knowledge on this subject is based on large case series, which are composed of “single” small renal masses, and patients with hereditary kidney cancer, such as von Hippel-Lindau disease, familial leiomyomatosis, and hereditary papillary renal cell carcinoma usually presenting with bilateral multiple tumors, constitute challenges [9, 11]. Taking into consideration that these patients develop renal tumors at younger ages and that their kidney pathologies are the major determinants of their prognosis, a great care has to be given in their management.

The incidence of multifocality of RCC ranges between 6 and 25% in published series [12, 13] The dilemma of the management of ipsilateral multiple tumors has recently been investigated by Lin et al. [11]. They compared the operative findings, renal functional outcomes, and intermediate-term cancer-specific survival of patients undergoing either laparoscopic partial nephrectomy or laparoscopic cryoablation and report similar results. However, the number of tumors they treated ranged between 2 and 3 per kidney, and the majority of tumors were <4 cm in size. Furthermore they report a median warm ischemia time of 36 minutes (range: 12–48), although they underline that the postoperative renal function was preserved in all patients in the intermediate term. Interestingly, they managed 2 patients in their series under cold ischemia only without vascular clamping but had to clamp the hilum because of a bleeding during tumor removal [11].

In the present patient, a total of 8 kidney tumors were removed with open NSS from two kidneys and the total surgical manipulation time just for tumor removals and renographies was 45 minutes per kidney, without vascular clamping. This time period is clearly higher than the universally accepted limits under warm ischemia. So even in highly experienced hands, using either open or robot assisted/laparoscopic approach, vascular clamping would increase the risk of renal function loss.

Recently, increasing number of papers are getting published regarding the safety of partial nephrectomy without renal hilar occlusion in selected cases [14]. Robot-assisted surgical techniques did clearly supply manipulative advantages, especially during reconstruction, when compared to pure laparoscopic techniques. However, multiple renal tumor removal for multifocal renal cancer, without renal hilar clamping, has not been reported with robot-assisted surgery yet.

In conclusion, we present the case that the nonvascular clamping open NSS, when used in patients with hereditary kidney cancer syndromes such as BHDS, minimizes the risk of renal function loss.

## References

[1] Birt AR, Hogg GR, Dube WJ (1977). Hereditary multiple fibrofolliculomas with trichodiscomas and acrochordons. *Archives of Dermatology*.

[2] Schaffer JV, Gohara MA, McNiff JM, Aasi SZ, Dvoretzky I (2005). Multiple facial angiofibromas: a cutaneous manifestation of Birt-Hogg-Dubé syndrome. *Journal of the American Academy of Dermatology*.

[3] Vincent A, Farley M, Chan E, James WD (2003). Birt-Hogg-Dubé syndrome: a review of the literature and the differential diagnosis of firm facial papules. *Journal of the American Academy of Dermatology*.

[4] Menko FH, van Steensel MA, Giraud S (2009). Birt-Hogg-Dubé syndrome: diagnosis and management. *The Lancet Oncology*.

[5] Fahmy W, Safwat AS, Bissada NK (2007). Multiple/bilateral renal tumors in patients with Birt-Hogg-Dubé syndrome. *International Urology and Nephrology*.

[6] Coleman JA, Russo P (2009). Hereditary and familial kidney cancer. *Current Opinion in Urology*.

[7] Delongchamps NB, Galmiche L, Eiss D (2009). Hybrid tumour ’oncocytoma-chromophobe renal cell carcinoma’ of the kidney: a report of seven sporadic cases. *British Journal of Urology International*.

[8] Pavlovich CP, Grubb RL, Hurley K (2005). Evaluation and management of renal tumors in the Birt-Hogg-Dubé syndrome. *Journal of Urology*.

[9] Steinberg AP, Kilciler M, Abreu SC (2004). Laparoscopic nephron-sparing surgery for two or more ipsilateral renal tumors. *Urology*.

[10] Becker F, Van Poppel H, Hakenberg OW (2009). Assessing the impact of ischaemia time during partial nephrectomy. *European Urology*.

[11] Lin YC, Turna B, Frota R (2008). Laparoscopic partial nephrectomy versus laparoscopic cryoablation
for multiple ipsilateral renal tumors. *European Urology*.

[12] Gohji K, Hara I, Gotoh A (1998). Multifocal renal cell carcinoma in Japanese patients with tumors with maximal diameters of 50 mm. or less. *Journal of Urology*.

[13] Whang M, O’Toole K, Bixon R (1995). The incidence of multifocal renal cell carcinoma in patients who are candidates for partial nephrectomy. *Journal of Urology*.

[14] White WM, Goel RK, Haber GP, Kaouk JH (2010). Robotic partial nephrectomy without renal hilar occlusion. *British Journal of Urology International*.

